# A Real-World Experience of the Short-Term Clinical Outcomes of Laparoscopic and Robotic-Assisted Ventral Hernia Repairs

**DOI:** 10.7759/cureus.81480

**Published:** 2025-03-30

**Authors:** Vivek Bindal, Dhananjay Pandey, Shailesh Gupta, Priyanka Agarwal, Akhil Dahiya, Divya Gupta, Usha D Bindal

**Affiliations:** 1 Department of GI, Minimal Access and Bariatric Surgery, Max Super Speciality Hospital, Ghaziabad, IND; 2 Department of GI, Minimal Access and Bariatric Surgery, Max Super Speciality Hospital, Delhi, IND; 3 Institute of Minimal Access, Bariatric and Robotic Surgery, Max Super Speciality Hospital, Ghaziabad, IND; 4 Department of Clinical and Medical Affairs, Intuitive Surgical, Sunnyvale, USA; 5 Department of Clinical Operations, Catalyst Clinical Services Pvt. Ltd., Delhi, IND; 6 Department of Biochemistry, Post Graduate Institute of Child Health, Noida, IND

**Keywords:** laparoscopic, pain, quality of life, robotic-assisted surgery, ventral hernia repairs

## Abstract

Background: This study aims to provide the first comparative real-world evidence of laparoscopic and robotic-assisted ventral hernia repairs performed in India, regardless of defect size or technique used.

Methods: The primary objective was to compare postoperative pain, analgesic use, and quality of life (QOL) between the two groups. Additionally, data on length of hospital stay, operating time, incidence of intra- and postoperative complications, re-admission rates, and use of tacks were collected and analyzed.

Results: The study included 290 cases: 111 (38.28%) in the robotic group and 179 (61.72%) in the laparoscopic group. Incisional hernias were more common in the robotic group (65 patients, 58.56%), while primary ventral hernias were more prevalent in the laparoscopic group (113 patients, 63.13%). The most common robotic repair approach was extended totally extraperitoneal Rives-Stoppa (eTEP-RS) with or without transversus abdominis release (TAR), performed in 75 (67.56%) cases. In the laparoscopic group, intraperitoneal onlay mesh (IPOM) plus was the most frequently performed procedure, used in 115 (64.25%) cases. The robotic group reported significantly lower pain levels after 6 hours, 24 hours, and 14 days following surgery. The number and duration of analgesic use were significantly reduced in the robotic group. Additionally, the robotic group had significantly better health perception scores. Compared to the laparoscopic group, the robotic group experienced significantly fewer postoperative complications (2 vs. 16; p = 0.013). Notably, the robotic group did not use tacks for mesh fixation, while tacks were employed in roughly 116 (64.8%) of the laparoscopic cases.

Conclusion: In real-world Indian settings, this study demonstrates the feasibility of robotic-assisted ventral hernia repairs, regardless of defect size or technique used.

## Introduction

Over 20 million hernia repair surgeries are performed annually worldwide, making it one of the most common general surgical procedures [1]. Primary and incisional ventral hernias are associated with significant morbidity and are frequently repaired using either open or laparoscopic techniques. According to the National Surgery Quality Improvement Program of the American College of Surgeons, the open approach has higher morbidity rates than minimally invasive laparoscopic techniques [2]. Laparoscopy offers advantages such as better identification of additional fascial defects or occult hernias and wider mesh coverage [3]. In real-world practice, the intraperitoneal onlay mesh (IPOM/IPOM plus) approach is the most commonly used laparoscopic method. However, it is associated with complications such as mesh-related adhesions, port-side hernias, and acute postoperative pain [4,5]. Although technically challenging, the laparoscopic transabdominal pre-peritoneal (TAPP) approach can overcome some of the challenges associated with the IPOM technique [6].

Robotic-assisted ventral hernia repairs have gradually been adopted over the last 15 years as an alternative to the challenges of both open and laparoscopic approaches [7,8]. Robotic-assisted surgery provides the surgeon with improved visibility, enhanced dexterity, easy deployment of preperitoneal mesh, improved endowrist movement, and better ergonomics. Using the robotic approach, the fascial defect can be repaired without the need for tacks or transfascial sutures, and mesh implantation can be performed in preperitoneal or retrorectus positions. In addition, it enables precise dissection in limited spaces [3]. Numerous real-world studies have compared open and laparoscopic approaches to robotic-assisted techniques. A large retrospective study comparing laparoscopic IPOM with robotic-assisted IPOM revealed that the robotic group had shorter hospital stays (0 vs. 1 day; p = 0.001) and fewer surgical site infections (5% vs. 14%; p = 0.001) [9]. Studies comparing the extended totally extraperitoneal (eTEP) and IPOM techniques for ventral and incisional hernia repair have shown that while the eTEP approach has a longer operating time, it results in a shorter hospital stay and significantly less postoperative pain [10]. However, clinical data on robotic-assisted ventral hernia repairs in India remains insufficient.

This study compared the short-term clinical outcomes of laparoscopic and robotic-assisted procedures, both routinely performed in real-world settings. We hypothesize that robotic-assisted repair results in better short-term clinical outcomes than laparoscopic repair.

## Materials and methods

The study was conducted at the Max Institute of Minimal Access, Bariatric and Robotic Surgery in Vaishali, Uttar Pradesh, India. A retrospective chart review was performed on consecutive patients who underwent ventral hernia repair via laparoscopic or robotic-assisted methods from January 2020 to October 2023. The inclusion criteria encompassed all types of ventral hernias, including primary, incisional, and recurrent cases, regardless of hernia size or patient-specific factors. Cases with incomplete data were excluded. Baseline characteristics, such as age, sex, BMI, comorbidities, and type of ventral hernia, were obtained from medical records. Intraoperative details, such as hernia defect size (length and width), the laparoscopic or robotic-assisted technique used for repair (e.g., IPOM, eTEP with or without transversus abdominis release (TAR), and transabdominal pre-peritoneal (TAPP)), mesh size, and the use of tacks for mesh fixation, were collected from surgical notes. The primary objective of this study was to compare laparoscopic and robotic-assisted ventral hernia repairs in terms of postoperative pain, QOL, and complication rates. The secondary objectives included evaluating differences in operative time, analgesic use, hospital stay, re-admission rates, and mesh fixation methods. No formal sample size calculation was performed, and the number of cases included was determined by the data collection period. Additionally, no adjustments for confounders were made, and no imputation was performed for missing data.

The primary outcome measures were pain and QOL in both groups. Pain was assessed using a numeric rating scale (NRS) with a range of 0-10 [11]. NRS data was collected at 6 hours, 24 hours, and 14 days after the surgery. In addition to the NRS, the number of analgesics taken and the duration of analgesic use (from surgery to discontinuation) were recorded. QOL scores were measured using the EuroQol 5D 3-Level Classification System (EQ-5D-3L) on day 14 following surgery [12]. The EQ-5D-3L descriptive system includes five dimensions: mobility, self-care, usual activities, pain/discomfort, and anxiety/depression. Each dimension is assessed on three levels: no issues, mild problems, and serious problems. The visual analog scale (EQ-VAS), ranging from 0 to 100 (with 0 representing the worst health and 100 representing the best health), was also used. The questionnaire data was collected on the 14th day after the surgery. Secondary outcome measures included operating room time, length of hospital stay, incidence of intraoperative and postoperative complications, re-admissions, and costs related to mesh and tacker used. All procedures (robotic-assisted or laparoscopic, IPOM, eTEP, eTEP with TAR) followed standard techniques recommended by surgeon societies. Robotic-assisted surgery was performed using the Da Vinci Xi Surgical System (Intuitive Surgical, Sunnyvale, CA, USA). All surgeries were performed by a single experienced surgeon.

Surgical technique

The initial entry into the left retrorectus space is made with an optical trocar in the upper abdomen, approximately 5-6 cm lateral to the midline. The retrorectus space is then expanded further using scope dissection.

Robotic arm

Two 8-mm secondary robotic trocars are placed just medial to the linea semilunaris, at the level of the umbilicus and the left lumbar region, carefully avoiding injury to neurovascular bundles. The robotic patient cart is docked from the right side of the patient. A crossover is performed into the opposite retrorectus space in the upper abdomen, followed by bilateral retrorectus dissection, which extends further caudally. During this process, the hernia sac is encountered and managed, with efforts made to preserve as much of the sac as possible. The posterior rectus sheath (PRS) in the midline is divided until the arcuate line, where the PRS merges with the transversalis fascia, leading into the space of Retzius and Bogros. Posteriorly, a tension-free closure of the PRS-peritoneal complex is performed using an absorbable barbed suture after reducing intra-abdominal pressure to 6-10 mmHg. The preserved sac is used to facilitate posterior closure, potentially eliminating the need for TAR in some cases. The anterior defect and linea alba are closed using a non-absorbable barbed suture. If a tension-free closure of the PRS-peritoneal complex is not feasible, TAR is performed. The dimensions of the created potential space are measured, and a macroporous polypropylene mesh is placed. Routine mesh fixation is not performed, and a drain is selectively placed as needed.

Laparoscopic arm

eTEP

After creating the retrorectus space on the ipsilateral side through blunt dissection, one 10-mm and one 5-mm secondary trocars are placed under needle guidance, just medial to the linea semilunaris. After the crossover, a 5-mm trocar is placed in the right retrorectus space under direct vision. Dissection in the retrorectus space and defect closure proceed in the same manner as described in the robotic technique. During the closure of the anterior rectus sheath, an additional 5-mm port may sometimes be placed at the level of the umbilicus in the right retrorectus space to assist in the cranial closure of the anterior rectus sheath. Thus, in the laparoscopic approach, 4-5 ports were used, whereas in the robotic approach, three ports were utilized.

IPOM

General anesthesia is administered first, and the patient is positioned supine with the left arm tucked alongside the body. The pneumoperitoneum is established at Palmer’s point in the left subcostal region. One 10-mm port and two 5-mm ports are inserted, typically positioned lateral to the left linea semilunaris. In some cases, one port is placed in the epigastrium, while the other two remain lateral to the left linea semilunaris. After port insertion, a 5-mm, 30-degree camera is used for visualization. The hernia contents are reduced, and the defect is identified. Closure of the defect is performed endoscopically using a barbed suture, with a transfascial approach occasionally employed. For additional analgesia, a TAP block is administered. The mesh placement site is marked on the skin using a surgical scale, based on the central point of the defect. Under strict aseptic conditions, the tissue-separating mesh is prepared, and transfascial sutures are applied at its four corners. The mesh is introduced through the 10-mm port, and the transfascial sutures are retrieved using a suture loop over a spinal needle. To secure the mesh, tackers are applied in two concentric circles. The 10-mm port is then closed transfascially, the pneumoperitoneum is released, and all ports are closed using subcuticular sutures.

The statistical analysis of quantitative variables was summarized as the arithmetic mean with standard deviation (SD). Categorical data were summarized using frequencies and percentages. The Pearson chi-square test or Fisher's exact test, as appropriate, was used to compare frequencies between groups. The differences in means between the robotic-assisted and laparoscopic groups were compared using the Student’s t-test. A two-sided p-value < 0.05 was considered statistically significant. Statistical analysis was performed using Stata 16.0 statistical software (StataCorp LLC, College Station, TX, USA).

## Results

Preoperative characteristics

A total of 290 cases were collected, 179 in the laparoscopic group and 111 in the robotic group. The robotic group had significantly higher weight and BMI compared to the laparoscopic group, although there were no significant differences in age or sex. Incisional hernias were more prevalent in the robotic group (58.56%), while primary ventral hernias were more common in the laparoscopic group (63.13%). The preoperative characteristics of the study population are presented in Table 1.

**Table 1:** Preoperative characteristics of the study population *Significant value. Statistical test: Pearson chi-square test or Fisher's exact test, Student’s t-test. NS: not significant, CAD: coronary artery disease, CKD: chronic kidney disease, CLD: chronic liver disease.

Variable	Robotic-assisted (N = 111)	Laparoscopic (N = 179)	p-value
Age, mean ± SD, year	52.65 ± 11.90	53.33 ± 11.68	NS
Sex, n (%)			
Female	71 (63.96)	120 (67.04)	NS
Male	40 (36.04)	59 (32.96)	NS
Weight, mean ± SD, kg	76.29 ± 14.90	71.67 ± 10.97	0.0028
BMI, mean ± SD, kg/m^2^	29.39 ± 5.76	28.13 ± 3.76	0.0252
Comorbidities, n (%)	88.3%	67.6%	-
Diabetes	24 (21.62)	35 (19.55)	-
Hypertension	44 (39.64)	58 (32.40)	-
Asthma	1 (0.90)	3 (1.68)	-
Obstructive sleep apnea	0 (0.00)	2 (1.12)	-
Thyroid	19 (17.12)	19 (10.61)	-
Obesity	0 (0.00)	2 (1.12)	-
Rheumatoid arthritis	1 (0.90)	1 (0.56)	-
CAD	2 (1.80)	0 (0.00)	-
CKD	3 (2.70)	1 (0.56)	-
Crohn’s	2 (1.80)	0 (0.00)	-
CLD	1 (0.90)	0 (0.00)	-
Parkinson’s	1 (0.90)	0 (0.00)	-
Type of ventral hernia, n (%)			
Incisional	65 (58.56)	66 (36.87)	0.000*
Primary ventral	46 (41.44)	113 (63.13)	0.000*
Recurrent hernia, n (%)			
Yes	22 (19.82)	18 (10.06)	NS
No	89 (80.18)	161 (89.94)	NS

Operative techniques

The robotic group mainly used eTEP or different variants of eTEP (67.56% of cases). The most common technique in the laparoscopic group (64.25%) was IPOM plus, where a mesh was placed intraperitoneally and required tackers for fixation. The robotic group had longer and wider hernia defects compared to the laparoscopic group (p = 0.0000). A significantly longer operating room time was observed in the robotic group. Additionally, the difference in length of hospital stay (2.42 vs. 2.13 days; p = 0.0014) was statistically significant but clinically marginal. Table 2 contains details about the operative techniques used in the study population.

**Table 2:** Operative techniques *Significant value. Statistical test: Pearson chi-square test or Fisher's exact test, Student’s t-test. eTEP-RS: extended totally extraperitoneal Rives-Stoppa, B/L: bilateral, TAR: transversus abdominis release, IPOM: intraperitoneal onlay mesh, TAPE: transabdominal partial extra-peritoneal, TAPP: transabdominal pre-peritoneal, TARUP: transabdominal retromuscular umbilical prosthetic hernia repair, INR: Indian rupee.

Variable	Robotic-assisted (N = 111)	Laparoscopic (N = 179)	p-value
Type of ventral hernia repair, n (%)			
eTEP-RS	47 (42.34)	17 (9.50)	0.0000*
eTEP-RS, B/L TAR	3 (2.70)	9 (5.03)	0.3329
eTEP-RS, bottom-up TAR	1 (0.90)	0 (0.00)	0.2036
eTEP-RS, left TAR	4 (3.60)	7 (3.91)	0.8931
eTEP-RS, right TAR	20 (18.02)	14 (7.82)	0.0087*
IPOM plus	13 (11.71)	115 (64.25)	0.0000*
TAPE	0 (0.00)	1 (0.56)	0.4297
TAPP plus	20 (18.02)	14 (7.82)	0.0087*
TARUP	2 (1.80)	2 (1.12)	0.6295
TARUP, Left TAR	1 (0.90)	0 (0.00)	0.2036
Total operating room time, mean ± SD, min	123.04 ± 60.28	100.91 ± 63.36	0.0036*
Length of hernia, mean ± SD, cm	8.22 ± 5.30	3.53 ± 2.87	0.0000*
Width of hernia, mean ± SD, cm	6.14 ± 3.75	2.80 ± 1.80	0.0000*
Length of mesh, mean ± SD, cm	24.14 ± 6.77	18.57 ± 5.87	0.0000*
Width of mesh, mean ± SD, cm	18.77 ± 5.15	17.56 ± 5.09	0.0518
Mesh Location, n (%)			
Intraperitoneal	13 (11.71)	116 (64.80)	0.0000*
Preperitoneal	21 (18.92)	14 (7.82)	0.0048*
Retrorectus	77 (69.37)	49 (27.37)	0.0000*
Tacks used, n (%)			
Yes	0 (0.00)	116 (64.80)	-
No	110 (100.00)	63 (35.20)	-
Number of tacks used, mean ± SD	0.0	17.36 ± 2.16	-
Mesh cost, mean, INR	16040.41	34615.27	0.0000*
Tacker cost, mean, INR	0.00	31887.12	-
Length of hospital stay, mean ± SD, days	2.42 ± 0.77	2.13 ± 0.72	0.0014*

Operative outcomes

There were no conversions to open surgery in either group. Pain was significantly lower in the robotic group at 6 hours, 24 hours, and 14 days following surgery. Compared to the laparoscopic group, the robotic group has a significantly lower mean number of analgesics (p = 0.0018) and shorter duration of analgesic use (p=0.0017). The robotic group also showed significantly better QOL scores. Postoperative complications were significantly fewer in the robotic group (1.8% vs. 8.94%; p = 0.013). No re-admissions occurred in the robotic group, while two cases required re-admission in the laparoscopic group. The surgical outcomes of the study population are presented in Table 3.

**Table 3:** Operative outcomes *Significant value. QOL scale: EQ-5D-3L. Pain scale: numeric rating scale. Statistical test: Pearson chi-square test or Fisher's exact test, Student’s t-test. UTI: urinary tract infection, AKI: acute kidney injury, QOL: quality of life.

Variable	Robotic-assisted (N = 111)	Laparoscopic (N = 179)	p-value
Postoperative complications, n (%)	2 (1.80)	16 (8.94)	0.013*
Bruising at port site	0 (0.00)	3 (1.68)	
Intestinal obstruction	0 (0.00)	1 (0.56)	
Retention of urine	0 (0.00)	2 (1.12)	
Seroma	1 (0.90)	7 (3.91)	
UTI	0 (0.00)	3 (1.68)	
AKI and chest infection	1 (0.90)	0 (0.00)	
Clavien-Dindo classification of postoperative complications, n (%)			
Grade I	1 (0.90)	13 (7.26)	0.025*
Grade II	1 (0.90)	3 (1.68)	-
Number of analgesics used per day before discharge, mean ± SD	4.73 ± 1.52	5.40 ± 1.90	0.0018*
Pain score, mean ± SD			
6 hours post-surgery	5.37 ± 1.14	7.03 ± 1.45	0.0000*
24 hours post-surgery	3.71 ± 1.07	5.27 ± 1.34	0.0000*
14 days post-surgery	2.04 ± 0.76	3.96 ± 1.31	0.0000*
QOL score, mean ± SD			
14 days post-surgery	86.34 ± 5.93	80.59 ± 4.90	0.0000*
Length of analgesic usage, mean ± SD, days	5.02 ± 2.23	5.84 ± 2.08	0.0017*
Re-admission (within 30 days), n (%)	0 (0.0)	2 (1.12)	0.526
Reoperations, n (%)	0 (0.0)	0 (0.0)	-

## Discussion

Before 2019, ventral hernias were traditionally repaired using open or laparoscopic approaches. Robotic-assisted ventral hernia repair techniques have emerged over the past few decades to tackle the challenges of open and laparoscopic approaches [7,8]. The robotic-assisted technique has been shown to have a lower risk of blood loss, infections, and postoperative complications compared to open repairs [13,14]. It also offers the benefit of a shorter hospital stay [13,14]. Furthermore, compared to laparoscopy, the robotic-assisted technique has been reported to have a decreased risk of conversion to open surgery and intraoperative bowel injury [14]. Patient-reported outcomes, such as the time it takes to return to daily activities, have been found to improve in certain studies [13,14]. Incisional and primary ventral hernias significantly affect QOL. Incisional hernias resulting from open abdominal surgery have a major influence on health-related QOL and body image, according to van Ramshorst et al. [15]. According to Cheatham et al. [16], people who have incisional hernias suffer a major loss of their emotional, social, and physical well-being. Research shows that incisional and primary ventral hernia repair significantly improves both overall and hernia-related QOLs, establishing postoperative QOL as an important outcome variable for hernia patients [17-20]. Traditionally, there has been a high degree of both acute and chronic pain following minimally invasive incisional and primary ventral hernia repairs because transfascial sutures and mesh fixation with tackers are employed in these surgeries [17]. Conventional measures of outcome include operating time, length of hospital stay, complications, and recurrence.

Our study examined pain, QOL, and other perioperative outcomes associated with robotically assisted and laparoscopic surgical procedures in real-world settings. To our knowledge, this is the first study of its kind in India to examine the short-term outcomes of laparoscopic and robotic-assisted ventral hernia repairs. A systematic review and meta-analysis from India assessed patient-reported outcome measures for laparoscopic and robotic incisional and primary ventral hernia repair procedures [13]. The review covered eight studies, comprising six cohort studies and two randomized controlled trials with a total of 41,205 patients. The robotic group demonstrated a statistically significant advantage when it came to recurrence rates, returning to work, and resuming everyday tasks. The two groups had similar lengths of hospital stay, re-admission rates, postoperative pain, QOL, and patient satisfaction. For postoperative pain, the quality of the evidence was rated moderate; however, for QOL, length of stay, recurrence, and re-admission, it was rated low to very low.

In terms of pain, our study found that robotic surgery improved pain scores compared to laparoscopy at all three time points: 6 hours post-surgery (5.37 ± 1.14 vs. 7.03 ± 1.45; p = 0.0000), 24 hours post-surgery (3.71 ± 1.07 vs. 5.27 ± 1.34; p = 0.0000), and 14 days post-surgery (2.04 ± 0.76 vs. 3.96 ± 1.31; p = 0.0000). The robotic group had significantly lower requirements for analgesics (4.73 ± 1.52 vs. 5.40 ± 1.90; p = 0.0018) than the laparoscopic group. Robotic-assisted eTEP repair allows for repair in the retrorectus plane without the need to enter the peritoneum and eliminates the requirement for mesh fixation using tackers. As a result, there is less acute postoperative pain and a lower need for analgesics. The robotic-assisted intraperitoneal approach has been compared with the laparoscopic intraperitoneal approach in most interventional studies in the literature. Therefore, these studies have not indicated a significant reduction in pain among the robotic group. However, in our study, eTEP was the predominant procedure in the robotic group, while IPOM was more common in the laparoscopic group. Naturally, this study is inclined to show less pain in the robotic arm due to the choice of procedure. Two randomized clinical trials that examined pain scores found an improvement in pain scores in the robotic group, around postoperative day 30, compared to laparoscopic techniques [13,21,22]. In terms of the standardized mean difference, the robotic group was more favorable (-1.42, 95% CI, -1.74 to -1.10). In addition, the robotic group reported a lower level of pain than the laparoscopic group. With a standard mean difference of -0.48 (-0.68, -0.28), it was clear that the robotic group had less pain after surgery. These findings are consistent with those from our study. Some studies have found improvement in postoperative pain when laparoscopic eTEP was compared to laparoscopic IPOM [23,24]. However, as stated earlier, laparoscopic eTEP is an extremely challenging technique even with the aid of an expert surgeon [6].

As with pain, the robotic group in our study showed a significant improvement in QOL VAS scores (86.34 ± 5.93 vs. 80.59 ± 4.90; p = 0.0000). The EQ-5D-3L was used to assess the patient's overall state of health and well-being on day 14. The patient's long-term QOL scores were not assessed, so these findings only show short-term QOL improvements. Our QOL findings cannot be compared to other studies because the literature only compares robotic-assisted IPOM to laparoscopic IPOM. The QOL score in these IPOM studies was only reported by a handful of individuals, and the robotic group was either comparable or superior [13,25]. According to the results of our study, the robotic group experienced significantly fewer postoperative complications (1.8% vs 8.94%; p = 0.013). These results are not in line with the literature and are most likely caused by dissection in the retrorectus space instead of intraperitoneal dissection.

The risk of hernia recurrence in the robotic group was significantly lower than in the open and laparoscopic groups, according to a meta-analysis of 23 studies [26]. However, we found no reoperations and a comparable re-admission rate within 30 days of surgery in both groups. Moreover, neither the robotic nor the laparoscopic procedures were converted to an open approach. The authors also conducted an explorative analysis of costs related to mesh in both groups. The eTEP approach in the robotic group significantly reduced the cost of mesh. Furthermore, mesh fixing led to significant cost savings by eliminating the requirement for tackers.

In this study, we have consciously compared the two groups regardless of hernia defect size and technique used. The decision to do this comparison was aimed at generating real-world data that may help Indian surgeons and healthcare administrators make a holistic decision related to ventral hernia repair. Although it is challenging to compare IPOM plus with retromuscular robotic techniques due to their technical differences, the results are interesting. Despite the greater complexity of robotic techniques, they still achieve favorable outcomes compared to laparoscopic techniques. Our group also intends to publish findings by comparing various techniques independently.

Strengths and limitations

Strengths

The fact that this study is the first of its kind in India to compare laparoscopic and robotic-assisted ventral hernia repairs is one of its merits. This is also one of a small number of real-world studies that compare laparoscopic IPOM ventral hernia repairs with a robotic-assisted eTEP approach. The study included a single expert surgeon who performed all the surgeries, ensuring uniformity in the operating techniques.

Limitations

The key limitations of this study include the fact that all procedures were performed by a single surgeon, which may limit its generalizability. Future studies should address inter-surgeon variability to enhance broader applicability. Additionally, long-term recurrence rates were not assessed due to the retrospective design and lack of long-term follow-up data, which are critical for evaluating the success of hernia repair. The study also does not provide an independent analysis of specific techniques (e.g., eTEP vs. eTEP/TAPP or IPOM vs. IPOM) or the size of the ventral hernia defect. Although robotic-assisted surgery incurs additional costs due to robotic disposables, it offsets expenses by eliminating the need for dual mesh and tackers required in laparoscopic ventral hernia repairs. This is because the robotic platform enables mesh placement outside the peritoneal cavity in a more predictable and replicable manner. Furthermore, given the baseline differences between the two groups, propensity score matching or multivariate adjustments were not conducted to control for confounders.

## Conclusions

In conclusion, the comparison between laparoscopic and robotic-assisted ventral hernia repairs in India highlights several notable benefits of the robotic-assisted approach. Robotic-assisted ventral hernia repairs significantly reduce pain, analgesic usage, and postoperative complications in Indian settings compared to laparoscopic procedures. While robotic-assisted surgery appears to be superior in terms of pain and QOL, the lack of long-term follow-up prevents assessment of recurrence rates and cost-effectiveness. Further studies with larger sample sizes, long-term follow-up, and cost-benefit analyses are needed to optimize robotic-assisted ventral hernia repair in India.
